# Long-term follow-up of acute porphyria in female patients: Update of clinical outcome and life expectancy

**DOI:** 10.1016/j.ymgmr.2022.100842

**Published:** 2022-02-02

**Authors:** Katrin Baumann, Raili Kauppinen

**Affiliations:** a Helsinki University Hospital, Department of Medicine, Finland; b Helsinki University Hospital, Department of Obstetrics and Gynecology, Finland

**Keywords:** Acute hepatic porphyria, Acute intermittent porphyria, Variegate porphyria, Hereditary coproporphyria, Acute attack, Primary liver cancer

## Abstract

**Background:**

Acute hepatic porphyria includes four inherited disorders caused by partial deficiencies of enzymes related to the heme biosynthesis. Clinical manifestations include acute attacks, occurring mainly among female patients. This study describes the diversity of acute symptoms, changes in triggering factors and life expectancy among female patients during the past five decades.

**Methods:**

107 Finnish female patients were enrolled into a retrospective, longitudinal study during 2015. Clinical, biochemical and genetic data was obtained from the medical reports, registry data and a questionnaire designed for the study. Causes of death were studied in additional 32 female patients.

**Results:**

Of the 43 patients with hospitalization, 33% had non-complicated, 35% prolonged and 28% severe attacks with no correlation with the disease-causing mutation. Of the deceased patients, 31% died of an acute attack during 1957–1979. Thereafter the incidence and severity of acute attacks have decreased substantially. 55% of the subjects reported acute symptoms (dysautonomia and mental symptoms) without hospitalization, 29% had porphyria symptoms >10 times, and 23% within the last year. Despite 22% of the female patients had died of primary liver cancer, the life expectancy increased more than 10 years during the follow-up, and did not differ from the normal population at present.

**Conclusions:**

The incidence of acute attacks requiring hospitalization has decreased, but more than half of the female patients reported acute symptoms affecting their well-being. Symptoms are currently triggered by hormonal changes and weight loss emphasizing the importance of early recognition and active management to avoid disease exacerbation. Death due to primary liver cancer is common and should be screened regularly.

## 1 Introduction

Acute hepatic porphyria (AHP) includes four inherited disorders caused by partial deficiencies of enzymes related to the heme biosynthesis [1]. Clinical manifestations include acute attacks comprising acute autonomic and peripheral neuropathy, and encephalopathy [2,3]. This is caused by the acute rise of neurotoxic porphyrin precursors, δ-aminolevulinic acid (DALA) and porphobilinogen (PBG) in the circulation [4]. Various exogenous and endogenous factors which up-regulate the rate-limiting ALA synthase-1 (ALAS1) in the liver, induce attacks [[5], [6], [7]].

AHP includes acute intermittent porphyria (AIP, MIM#176000), variegate porphyria (VP, MIM#176200) and hereditary coproporphyria (HCP, MIM#121300) with autosomal dominant pattern of inheritance and heterogeneous genetic background. ALA-dehydratase deficiency porphyria (ADP, MIM#125270) is extremely rare and inherited recessively.

AHP has been reported worldwide. The prevalence of AIP varies from 1 to 10:100000 in different countries, and the estimated prevalence of the disease related mutations in the general population is 1:1299–1700 [[8], [9], [10]]. The incidence and prevalence in Europe are 0.13 and 5.4 per million inhabitants for AIP and 0.08 and 3.2 per million inhabitants for VP. The incidence for HCP has been calculated at 0.0195 per million inhabitants [10].

Wide variation in penetrance (10–42%) depends on the clinical presentation studied [[10], [11], [12]]. Diversity of clinical presentations even within families with the same genotype indicates that individual risk for acute symptoms depends on sex, other modifying genes and precipitating factors [9,11].

Inheritance of the mutations causing AHP is equal between sexes, but female patients are more frequently affected than male [[12], [13], [14]]. Acute symptoms manifest during childbearing age, commonly precipitated by metabolic and hormonal changes during the luteal phase of the menstrual cycle [5,6].

Acute porphyria is typically diagnosed at 20–40 years of age [11,15,16]. Diagnosis is often delayed by several years, although it could be easily confirmed with measurement of porphyrin metabolites [11,13]. Genetic screening of at–risk relatives and genetic counseling is key for patients to remain asymptomatic [11,17].

Early diagnosis and accurate treatment have decreased the morbidity and mortality of acute attacks [11,12,18,19]. Treatment with hematin quickly down regulates the expression of ALAS1 and is currently the only approved therapy for an attack [16,20]. Symptomatic treatment, including pain killers, and high-dose glucose infusions can be administered with the elimination of porphyrinogenic drugs and other triggering factors [21,22].

AHP has been associated with the long-term complications such as hypertension, chronic kidney disease and liver cancer [6,23,25]. A few patients have developed chronic neuropathy and encephalopathy after recurrent attacks [13,15,26,27].

The object of this study was to obtain further insight on the natural course of AHP at present among female patients. We conducted a retrospective, longitudinal study on 107 female patients from the Finnish porphyria patient registry to describe the changes in the clinical manifestations and triggering factors during the follow-up. Furthermore, we investigated the mortality and causes of death among Finnish female AHP patients in this cohort and the incidence in female patients.

## 2 Materials and methods

### 2.1 Patients

107 patients were enrolled from the Helsinki University Hospital (HUH) porphyria patient registry kept since 1966. Clinical, biochemical and genetic data was obtained from the medical reports, registry data and a novel comprehensive questionnaire designed for the study.

The diagnosis of AHP was confirmed by disease–causing mutations in HMBS, PPOX or CPOX genes. Biochemical data was available for 100 subjects enrolled [11].

The study group was set to include women of 14–85 years of age. Of the 182 living female subjects, 164 patients were within this age group. 75% of the subjects traced were enrolled for the study during 2015. 30 female patients (24 AIP and 6 VP) in the same age group had deceased during 1957–2018. The incidence for female patients during 1950–2019 was calculated using the HUH porphyria patient registry. Informed consent was obtained from all study patients, and from their guardians in the case of adolescents. The Ethical Committee of the Department of Medicine, HUH, approved the study protocol.

36 subjects (24 AIP, 12 VP) did not participate and 21 could not be traced, primarily due to living abroad. The median age was 54 years in 2015 and 39% of them had been hospitalized due to attacks, which did not differ substantially from the group studied.

### 2.2 Acute attack

Acute attack was defined as a combination of episodic symptoms of pain, autonomic and peripheral neuropathy together with CNS involvement in patients with at least a 4-fold increase in urinary levels of porphyrin precursors. Acute symptoms were similar, but treated at home with symptomatic treatment, and commonly no biochemical analysis was done. Acute symptoms persisted several days and resolved within a week [1].

Acute attacks were classified into three categories according to symptom severity associated with prognosis: [28]

I. Non-complicated attacks consisting of classical symptoms such as pain, autonomic neuropathy, mental symptoms and/or red urine.
II. Prolonged attacks involving, in addition to classical symptoms, sensorimotor neuropathy, encephalopathy and/or hyponatremia.
III. Severe attacks involving additionally impairment of consciousness, cardiac arrhythmias, bulbar palsy, hemi−/tetraplegia and/or respiratory failure.

Statistical calculations were performed with SPSS version 27 (2020). *P* < 0.05 was chosen as the level of statistical significance. Categorical variables were compared using Fishers exact test, and continuous variables with Student's *t*-test.

## 3 Results

### 3.1 Long-term follow-up

**Fig. 1:**
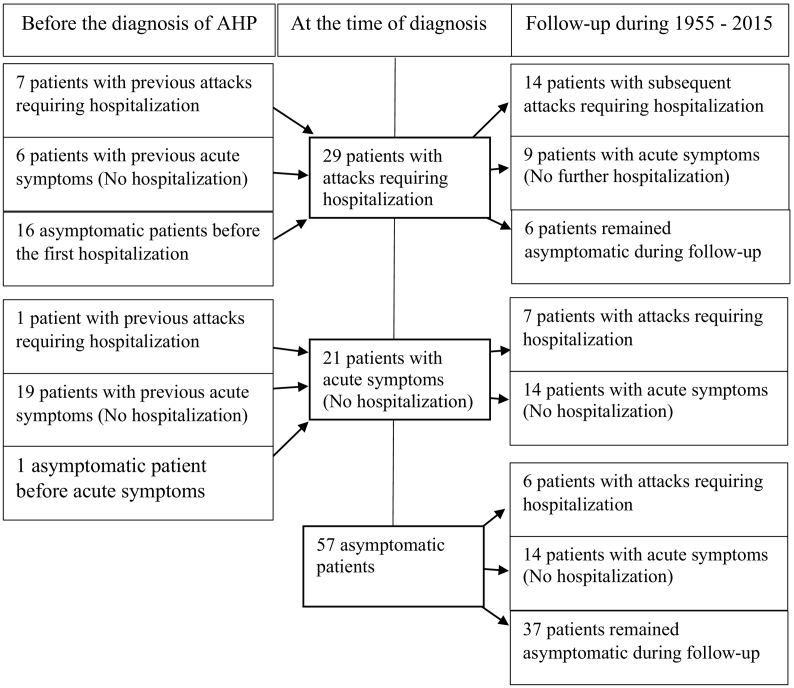
AHP manifestations at the time of the diagnosis and during the long-term follow-up.

The study group consisted of 107 female subjects of whom 65 were diagnosed with AIP, 40 with VP and 2 with HCP during 1955–2015 at a median age of 24 years (Table 1, Fig. 1).

**Table 1:** Long-term follow-up of 107 female AHP patients.

	AIP *n* = 65	VP *n* = 40	HCP *n* = 2	Total *n* = 107
Current age (2015)	50 (15–84)	57 (18–85)	52 (37–67)	54 (15–85)
Age at diagnosis	21 (0–47)	33 (13–76)	25 (16–33)	24 (0–76)
Mode of presentation (%)				
Acute attacks (hospitalization)	26 (40)	15 (38)	2 (100)	43 (40)
Acute symptoms solely (no hospitalization)	18 (28)	9 (23)	0	27 (25)
Cutaneous symptoms		21 (53)	0	21 (20)
Asymptomatic	21 (32)	7 (18)	0	28 (26)
Median age at symptom onset and end (range, years)				
Onset of acute symptoms	23 (10–57)	25 (11–48)	16, 33	23 (10–57)
Onset of cutaneous symptoms		26 (10–48)		
First hospitalization	25 (15–57)	31 (21–44)	16, 34	27 (15–57)
Last hospitalization	27 (16–57) *n* = 23	37 (21–64) *n* = 15	16, 34 *n* = 2	29 (16–64) *n* = 40
End of acute symptoms	33 (14–57) *n* = 24	40 (15–76) *n* = 16	16 *n* = 1	37 (14–76) *n* = 41
Time period of acute symptoms if ended (years)	5 (<1–43)	11 (<1–54)	<1	8 (<1–54)
Patients with acute symptoms during the past year (%)	17/44 (39)	7/24 (29)	1 / 2 (50)	25/70 (36)

**Fig. 2:**
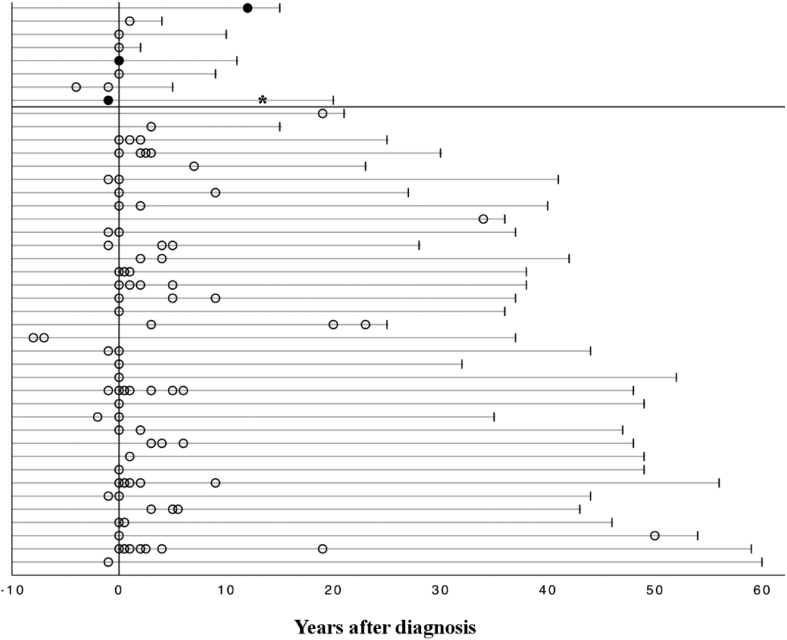
Distribution of first and subsequent attacks with hopsitalization among 43 patients during the follow-up. Patients in Groups A (above) and B are separated with a vertical line. White circles indicate an attack; black dots represent patients with recurrent attacks; *indicates the point of liver transplantation.

Of the study group, 37% reported sporadic attacks, of whom 33% had persistently high excretion of urinary PBG (>10-fold) and DALA (>4-fold) in remission. The majority of them experienced 1–2 attacks (range 1–7 attacks) during a median span of 3 years (1–20 years), with a median 12 months (range 1–180 months) between the attacks (Fig. 2).

3 AIP patients had recurrent attacks (≥4 attacks per year) for more than 10 years (Fig. 2) and extremely high urinary levels of PBG (>30-fold) and DALA (>5-fold). One of them underwent liver transplantation, after which she became asymptomatic with normal porphyrin metabolism but recovery from muscle weakness and fatigue lasted for years. Two patients' symptoms were alleviated after 2 months and 2 years on givosiran (Givlaari®).

Of the subjects, 25% reported porphyria related acute symptoms but had never been hospitalized due to an attack. 7% of them had high urinary levels of PBG (>10-fold) and DALA (>4-fold) in remission. 35% reported no porphyria related symptoms during a median follow–up of 52 years (Fig. 2), of whom 19% had normal, and 5% high excretion of urinary PBG (>10-fold) and DALA (>4-fold).

#### 3.1.1 Acute attacks

A total of 105 attacks among 43 patients required hospitalization during 1954–2015. Detailed information was obtained from 74 attacks. Recurrent attacks requiring weekly hematin prophylaxis were excluded. Of the hospitalized patients, 33% (*n* = 14) manifested with non-complicated attacks (see methods, I), 35% (*n* = 15) had prolonged attacks (II) and 28% (*n* = 12) severe attacks associated with a poor prognosis (III). Attack severity did not correlate with the disease-causing mutation.

Severe attacks (III) were commonly the first attack requiring hospitalization, and misdiagnosis led to attack exacerbation. Despite earlier diagnosis of AHP, mistreatment led to a severe attack in 4 cases. None of the 12 patients had multiple severe attacks, but 10 remained symptomatic and 8 of them were re-hospitalized during the follow-up.

**Fig. 3:**
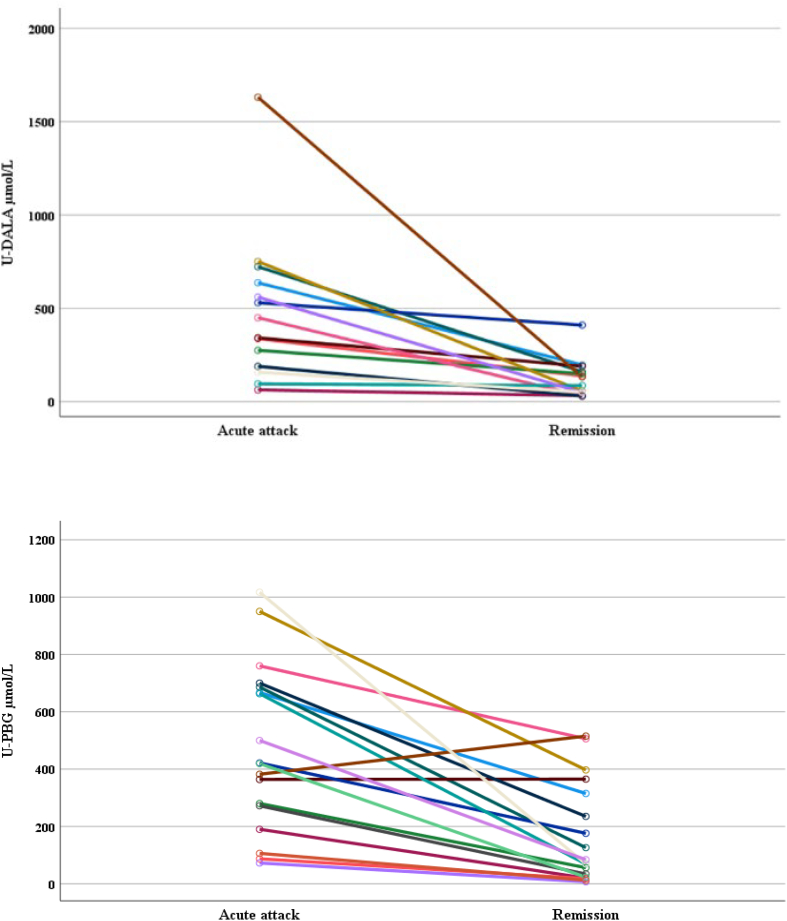
Quantitative DALA (*n* = 14) and PBG (*n* = 18) measures in spot urine samples during remission and acute phase among AHP patients. DALA = delta–aminolevulinic acid (<34 μmol/L), PBG = porphobilinogen (<9 μmol/L).

PBG and DALA levels were measured from urine samples showing wide variation (Fig. 3). In remission, the median PBG level was 76.5 μmol/L (range 7–515 μmol/L; AIP 213 μmol/L, range 20–515 μmol/L; HCP 18 μmol/L; VP 38 μmol/L, range 7–84 μmol/L) and DALA 86 μmol/L (30–410 μmol/L; AIP 136 μmol/L, range 30–410 μmol/L; HCP 71 μmol/L; VP 66 μmol/L, range 30–133 μmol/L), and during attacks PBG 418 μmol/L (66–1017 μmol/L; AIP 471 μmol/L, range 56–910 μmol/L; HCP 87 μmol/L; VP 401 μmol/L, range 72–1017 μmol/L) and DALA 396 μmol/L (63–1631 μmol/L; AIP 440 μmol/L, range 41–993 μmol/L; VP 635 μmol/L, range 158–1631 μmol/L).

The most common symptoms during attacks were abdominal pain, autonomic neuropathy and peripheral neuropathy, of which sensory neuropathy was reported in 65% and motor neuropathy in 28% of the attacks (Table 2). Mental symptoms were common but transient. AHP subtype, age, attack severity and frequency showed no correlation with patients' mental symptoms or perception of symptom severity.

**Table 2:** Symptoms and signs during acute attacks and acute symptoms.

	Among 27 patients with acute symptoms solely (no hospitalization) (%)	In 74 acute attacks resulting in hospitalization (%)	Among 70 symptomatic patients (%)
**Abdominal pain**	25 (93)	68 (92)	**66 (94)**
**Autonomic neuropathy**			**67 (96)**
Constipation	8 (19)	42 (57)	37 (53)
Nausea / Vomiting	11 (41)	49 (66)	42 (60)
Sinus tachycardia	2 (7)	36 (49)	29 (41)
Hypertension	0	23 (31)	22 (31)
Bladder paresis	0	6 (8)	8 (11)
**Peripheral neuropathy**			
**Motor neuropathy**			**23 (33)**
Muscle weakness	4 (15)	20 (27)	22 (31)
Respiratory paralysis	0	2 (3)	4 (6)
Bulbar palsy	1 (4)	4 (5)	6 (9)
**Sensory neuropathy**			**55 (79)**
Pain in the limbs	16 (59)	38 (51)	44 (63)
Back pain	13 (52)	35 (47)	34 (49)
Sensory disturbances	2 (7)	7 (9)	14 (20)
**CNS involvement**			
**Mental symptoms**	18 (67)	36 (49)	44 (63)
Insomnia	6 (22)	19 (26)	20 (29)
Melancholy	8 (30)	13 (18)	20 (29)
Fatigue	4 (15)	25 (34)	18 (26)
Anxiety	4 (15)	13 (18)	16 (23)
Confusion	2 (7)	7 (19)	10 (14)
Restlessness	2 (7)	11 (15)	9 (13)
Irritability	1 (4)	13 (18)	5 (7)
Decreased consciousness	0	5 (7)	10 (14)
Seizures	0	4 (5)	4 (6)
Hemi–/Tetraplegia	0	5 (7)	5 (7)
**Hyponatremia**	1 (4)	22 (30)	**19 (27)**
**Red urine color**	2 (7)	35 (47)	**29 (41)**

#### 3.1.2 Acute symptoms

Of the study group, 55% (*n* = 59) reported abdominal pain, mental symptoms and dysautonomia, which did not lead to hospitalization. During the follow-up, 24% (*n* = 14) reported symptoms 2–4 times, 24% (n = 14) 4–10 times, 7% (*n* = 4) 11–20 times, and 46% (*n* = 27) >20 times.

32 subjects had both acute attacks and non-hospitalized acute symptoms. 27 subjects had never been hospitalized due to AHP, but reported similar acute symptoms (Table 2). 11 patients required hospitalization in each acute attack. Acute symptoms and attacks manifested during a median span of 8 years with a wide range of intervals (<1 month to 54 years).

#### 3.1.3 Chronic symptoms

Of the subjects, 5.6% (*n* = 6; 5 AIP and 1 VP) reported chronic symptoms. Chronic symptoms manifested as persisting sensory neuropathy: tingling and sensory loss in lower extremities, pain in lower and upper extremities, back and abdomen. Chronic motor neuropathy manifested as muscle weakness, low endurance together with a sensation of slow recovery from physical exercise especially in lower extremities. Chronic mental symptoms were insomnia, fatigue and irritability.

All subjects with chronic symptoms had a history of multiple acute attacks. Two patients had recurrent attacks (>100 attacks during follow-up) and four patients mean four acute attacks (range 1–7 attacks) requiring hospitalization and additional acute symptoms. One patient became symptom free after 9 years during pregnancy. The mean urinary PBG excretion in remission was 270 μmol/L among AIP patients (*n* = 5, range 20–506 μmol/L) and DALA 197 μmol/L (*n* = 4, range 32–410 μmol/L).

### 3.2 Medication

Of the subjects, 62% (*n* = 66) had daily medications (sex hormones excluded) and 19% (*n* = 20) only occasionally. Medication for cardiovascular diseases, pain and allergies was frequent. At the time of reply, 34% of the patients (*n* = 36, median age 65 years, range 43–85 years of age) used medication for hypertension, of whom 69% had manifest AHP. The drugs used included diuretics, ACE inhibitors, ATR2, calcium channel, alpha and beta blockers.

Of the study group, 49% (*n* = 52) used pain killers. NSAID or acetylsalicylic acid were used by 41 subjects. Other pain-killers used were acetaminophen (paracetamol), codeine, tramadol, pregabalin, and sumatriptan or zolmitriptan for migraine (*n* = 29).

Other drugs used regularly without porphyria symptoms were psychotropic medication, immunosuppressant, anti-inflammatory and anti-cancer medication. Antiepileptic medication such as levetiracetam and lamotrigine caused no porphyria symptoms. Of the patients, 61% (45 symptomatic, 20 asymptomatic) used supplements, and none of them reported porphyria symptoms.

### 3.3 Subjects' perception of symptom severity

Subjects' perception of symptom severity correlated with the classification of acute manifestations of AHP (*p* < 0.001). Attacks were regarded as severe by 28% or extremely severe by 58% of the patients, the majority of whom had more than one attack. Patients with solely acute symptoms regarded their symptoms mild (15%), moderate (54%), severe (23%) or extremely severe (8%).

### 3.4 Changes in the natural history of AHP during the last six decades

Subjects were divided into two groups according to their current age to elucidate the changes in AHP manifestations with time (Table 3). The proportion of symptomatic patients was equal in both groups. The number of patients requiring hospitalization decreased significantly among AIP patients (Table 3), but not among VP patients. Acute symptoms were still frequent in both diseases. All patients with recurrent attacks were in Group A.

**Table 3:** Comparison of acute manifestations among Groups A (median 36 years, range 15–49 years) and B (median 64 years, range 50–85 years).

	AIP		VP		HCP		TOTAL	
	**A**	**B**	**A**	**B**	**A**	**B**	**A**	**B**
**No. of patients**	32	33	11	29	1	1	44	63
**Follow up fertile years**	631	1221	269	1073	25	37	925	2331
**Asymptomatic (%)**	14 (44)	7 (21)	3 (27)	13 (45)	0	0	17 (39)	20 (32)
***p***	0.066		0.473				0.537	
**Symptomatic (%)**	18 (56)	26 (79)	8 (73)	16 (55)	1	1	27 (61)	43 (68)
***p***	0.052		0.312				0.461	
**Acute symptoms solely (%)** (no hospitalization)	14 (44)	4 (12)	5 (45)	4 (14)	0	0	19 (43)	8 (13)
***p***	0.004*		0.032*				0.0004*	
**No. of patients with hospitalization (%)**	4 (13)	22 (67)	3 (27)	12 (41)	1	1	8 (18)	35 (56)
***p***	<0.001*		0.486				<0.001*	
**No. of patients with recurrent attacks**	3	0	0	0	0	0	3	0
**Mean of acute attacks per hospitalized patient**	1	2.45 ± 1.74	1.33 ± 0.58	2.42 ± 1.31	1/1	1/1	1.2 ± 0.45	2.4 ± 1.58
***p***	0.422		0.064				0.002*	
**Ratio of acute attacks per patient during fertile years**^**1**^	0.0013± 0.0071	0.0442± 0.0496	0.0112± 0.0202	0.0270± 0.0396	–	–	0.0049± 0.0137	0.0360± 0.0453
***p***	<0.001*		0.106				<0.001*	
**No. of patients treated with hematin**	3	2	2	7	1	0	6^2^	9^3^

^1^Follow up fertile years, 12–49 years of age; ^2^Hematin treatment during >100 attacks (1994 – 2015); ^3^Hematin treatment during 21 attacks (1980 – 2013).

Hematin treatment (Panhematin® in USA, Normosang® in Europe) has been available in Finnish hospitals since 1980 and was administered in 35 of the 48 attacks (73%). Two patients with recurrent attacks received prophylactic heme infusions weekly or biweekly. Of the 43 patients with acute attacks, 15 individuals (35%) were treated with hematin. The number of patients treated with hematin did not increase with time.

### 3.5 Causes of death

32 female patients (25 AIP and 7 VP) deceased during 1957–2018. The causes of death were acute attack (8 AIP, 2 VP), primary liver cancer (6 AIP, 1 VP), kidney failure (1 AIP) and other causes not related to AHP (*n* = 14). The median age at death was 65 years (range 19–87 years): 55 years (range 19–87 years) for AIP patients, and 68 years (range 23–85 years) for VP, respectively. When deaths due to acute attacks were excluded, the median age at death was 74 years (range 43–87 years; AIP 73 years, range 43–87; VP 74 years, range 65–85 years), which did not differ from the life expectancy of age-matched general female population in Finland.

Of the patients, 31% died during an attack between years 1957–1979, at the median age of 25 years (AIP 25, VP 28 years of age, range 19–45 years) after an average 3.3 attacks (AIP 3.8, VP 1, range 1–9 attacks). Symptoms had started at the median age of 21 years (range 15–37 years). 7 patients were diagnosed during their first attack, 2 patients post mortem and one patient 6 years prior to her death, 2 years after symptom onset.

Of all patients, 22% died of primary liver cancer, at the median age of 77 years (range 68–83 years) during 2010–2018. 57% of them (*n* = 4/7) had a history of manifest AHP. Among AIP patients with primary liver cancer, urinary PBG levels in remission were increased 14-fold (mean 126 μmol/L, range 49–192 μmol/L) and DALA levels 3-fold (mean 88 μmol/L, range 35–161 μmol/L) when compared with reference values.

### 3.6 Triggering factors

Of the 105 attacks treated at hospital, one or several triggering factors were identified and reported in 47% of the attacks (*n* = 49). Infection (47%), medication (47%) and the luteal phase of the menstrual cycle (35%) were most common, and less commonly weight loss (18%), postpartum period (8%), and alcohol (8%). Infection, stress and medication were less common triggers in Group A (Table 4), but the impact of hormonal changes and weight loss prevailed.

**Table 4:** Comparison of triggering factors among Groups A and B.

	**Group A** (15–49 years) *n* = 44 (%)	**Group B** (50–85 years) *n* = 63 (%)	**Total** *n* = 107 (%)	***p***
Menstrual cycle	23 (52)	26 (41)	49 (46)	0.325
Medication	8 (18)	23 (37)	31 (29)	0.052
Infection	5 (11)	23 (37)	28 (26)	0.004*
Weight loss	9 (20)	15 (24)	24 (22)	0.815
Alcohol	6 (14)	17 (27)	23 (21)	0.150
Stress	3 (7)	15 (24)	18 (17)	0.034*
Pregnancy	2 (4)	6 (10)	8 (7)	0.466
Postpartum period	1 (2)	5 (8)	6 (6)	0.398
Tobacco	1 (2)	0	1 (1)	0.411

Of the patients, 29% associated acute symptoms with drugs (Table 4). Medications used before attacks were commonly known triggers, such as barbiturates (Group B: 6 patients) and sulfonamides (Group B: 7 patients), which are rarely used at present. In both groups, pivmecillinam used in urinary tract infections was reported as a precipitating factor (Group A: 1 patient, B: 2 patients). In addition, one attack was associated with high-dose valproate, combined with oxcarbazepine (Group A) and another with metamizole and pitofenone (Group B). Topiramate (*n* = 1), ambroxol (n = 1) and levonorgestrel – ethinyl oestradiol combination (n = 1) were self-reported as possible triggers not leading to hospitalization.

Use of tobacco products was reported by 36% of the subjects, 82% of whom were ex–smokers. Only one subject associated smoking with acute symptoms. Of all patients, 21% reported alcohol as a triggering factor (Table 4).

In 2015, 58% of the subjects were within the normal weight range (BMI 18.5–24.9), 35% overweight and 7% underweight. During the follow-up, 40% (*n* = 43) had lost weight (median 10 kg, range 0.5-30 kg) of whom 37% (*n* = 16) reported acute symptoms during weight loss and other 8 patients during fasting.

### 3.7 Incidence of acute attacks and symptoms during 1950–2019

A total of 348 AIP patients, 188 female and 160 male, have been diagnosed in Finland to date. 87 female patients (46%) had a history of attacks leading to hospitalization. 77 of them had their first attack between 1951 and 2018. Of 206 VP patients (114 female and 92 male), 33 female patients (29%) were hospitalized due to an attack, 27 of whom during 1952–2013.

**Fig. 4:**
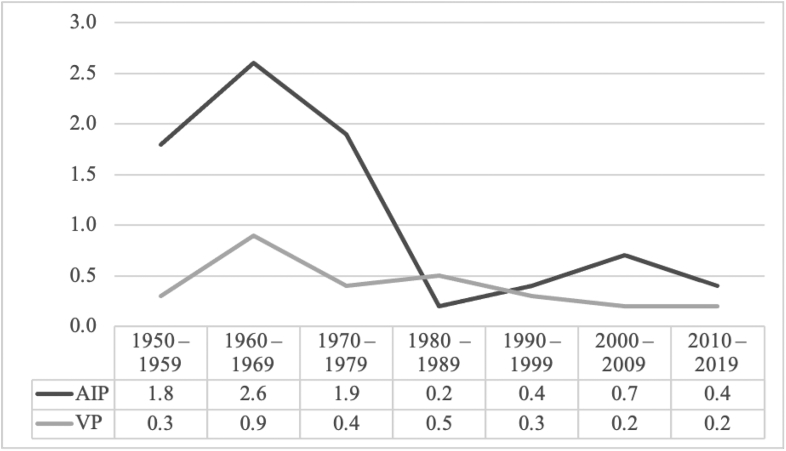
Incidence of acute attacks among female patients during 1950–2019.

The incidence of AIP and VP was based on the analysis of 77 AIP and 27 VP female patients with attacks during 1950–2019, and equaled 1.1 per million female inhabitants aged 15–44 years (reproductive age) for AIP and 0.4 for VP, respectively. A substantial decrease in the incidence of AIP could be seen in the 1980's which continued through the next four decades (Fig. 4).

## 4 Discussion

The object of this study was to provide up-dated insight on the clinical presentation, triggering factors and cause of death among female patients with AHP. We used the well-characterized Finnish patient registry and a comprehensive questionnaire to analyze the current state and long-term outcomes of AHP. A comparative study was designed to elucidate how current management, and avoidance of triggering factors has affected the mortality and morbidity of the patients.

In our study, more than half of the patients were symptomatic and hospitalized attacks represented only a small percentage of all acute symptoms. One third of the female patients had porphyria symptoms >10 times during the follow-up, and reported clinical manifestations within the last year. Patients considered their symptoms severe affecting their well-being dramatically [11,15,26,27]. Active phase of the disease lasted usually 5–10 years. This emphasizes the importance of active investigation and management of symptoms at the early phase to avoid disease exacerbation.

Half of the subjects reported acute symptoms during the luteal phase, which was the most common triggering factor especially among younger patients. Abdominal and back pain together with pain in the limbs were the most frequent symptoms showing a cyclic pattern, which was in accordance with other series [5,6].

A substantial decrease in the incidence of AHP could be seen since the 1980's. The role of infection and medication as triggering factors has diminished, but weight loss has remained. Our patients use various non-porphyrinogenic drugs according to the drug safety databases, encouraging the proper treatment of other diseases [7].

Despite the diagnosis of AHP, one third of the female patients died of an attack until 1980. Thereafter, recurrent attacks have become a phenomenon in 3% of the patients [15,27]. Liver transplantation, which normalizes porphyrin metabolism instantly, has been performed since 2002 and cured patients with recurrent attacks [29]. Givosiran, which is a small inhibitory RNA targeted to liver ALAS1 (Givlaari®), has diminished the frequency and severity of recurrent attacks [30].

After the development of hematin preparations in the 1980's, early treatment decreased attack mortality [20]. Genetic screening and counseling of at-risk relatives since the 1990's has decreased morbidity [11]. Death due to acute porphyria has become rare, and in our survey the life expectancy increased substantially during the follow up and does not differ from that of the normal female population at present.

Hepatocellular carcinoma is still a considerable cause of death among AHP patients and should be screened for after 50 years of age [1,31]. Not all patients with hepatoma had a history of manifest AHP but especially, if a patient becomes symptomatic at a later age, hepatoma should be excluded. The risk of hepatocellular carcinoma due to hepatitis virus or alcohol induced liver cirrhosis among AHP patients in Finland has been low [24]. Although one third of the patients, mainly symptomatic, used medication for hypertension, death of chronic kidney failure was rare [25].
